# Correction: Rhodium-catalysed selective C–C bond activation and borylation of cyclopropanes

**DOI:** 10.1039/d1sc90035h

**Published:** 2021-03-03

**Authors:** Yandong Wang, Jingyi Bai, Youqing Yang, Wenxuan Zhao, Yong Liang, Di Wang, Yue Zhao, Zhuangzhi Shi

**Affiliations:** State Key Laboratory of Coordination Chemistry, Chemistry and Biomedicine Innovation Center (ChemBIC), School of Chemistry and Chemical Engineering, Nanjing University Nanjing 210093 China shiz@nju.edu.cn yongliang@nju.edu.cn

## Abstract

Correction for ‘Rhodium-catalysed selective C–C bond activation and borylation of cyclopropanes’ by Yandong Wang *et al.*, *Chem. Sci.*, 2021, DOI: 10.1039/d0sc06186g.

The authors regret that there were a few errors in Fig. 3 of the original article. The correct version of Fig. 3 is shown below.

**Fig. 3:**
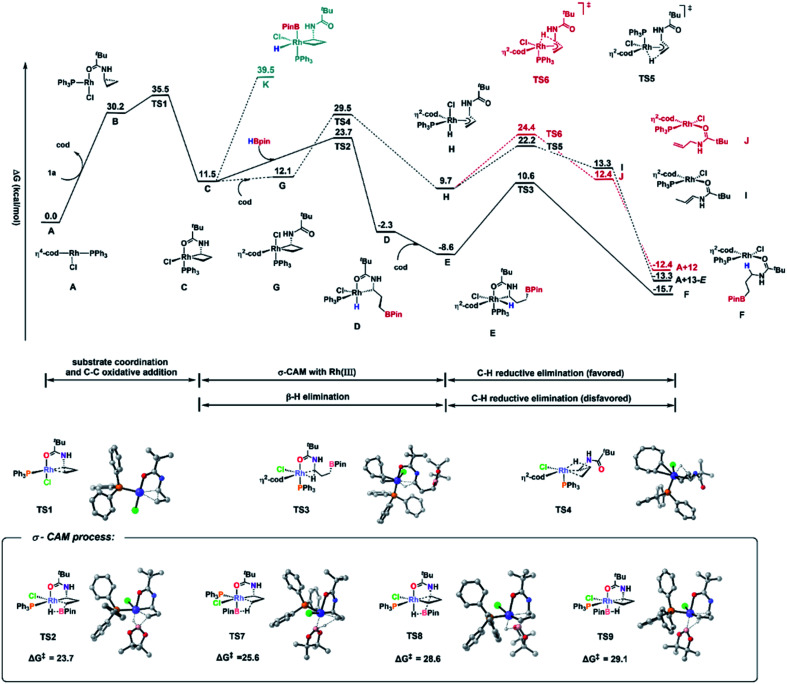
Free energy diagram of hydroboration of CPAs using PPh_3_ as the ligand and DFT-computed four transition states for the σ-CAM pathways. Energies are in kcal mol^−1^.

On pages 6–7 of the original manuscript, the sentences from “The enamide **I** is the kinetically favoured product” to the end of this paragraph should be corrected to “Finally, the catalytic species **A** is regenerated *via* dissociation of **12** or **13-*E*** from the Rh center. The enamide **13-*E*** is the kinetically favoured product, which supports the observation of **13** in Scheme 3A-3, where HBpin is not added.”

These changes do not alter the scientific conclusions of the manuscript.

The Royal Society of Chemistry apologises for these errors and any consequent inconvenience to authors and readers.

